# Association between Serum Cystatin C and Diabetic Foot Ulceration in Patients with Type 2 Diabetes: A Cross-Sectional Study

**DOI:** 10.1155/2016/8029340

**Published:** 2016-09-07

**Authors:** Jie Zhao, Wuquan Deng, Yuping Zhang, Yanling Zheng, Lina Zhou, Johnson Boey, David G. Armstrong, Gangyi Yang, Ziwen Liang, Bing Chen

**Affiliations:** ^1^Department of Endocrinology, Diabetic Foot Center, Southwest Hospital, Third Military Medical University, Chongqing 400038, China; ^2^Department of Podiatry, Singapore General Hospital, Singapore 169608; ^3^Department of Surgery, Southern Arizona Limb Salvage Alliance (SALSA), University of Arizona Health Sciences Center, Tucson, AZ 85724, USA; ^4^Department of Endocrinology, Second Affiliated Hospital, Chongqing Medical University, Chongqing 400010, China

## Abstract

Serum cystatin C (CysC) has been identified as a possible potential biomarker in a variety of diabetic complications, including diabetic peripheral neuropathy and peripheral artery disease. We aimed to examine the association between CysC and diabetic foot ulceration (DFU) in patients with type 2 diabetes (T2D). 411 patients with T2D were enrolled in this cross-sectional study at a university hospital. Clinical manifestations and biochemical parameters were compared between DFU group and non-DFU group. The association between serum CysC and DFU was explored by binary logistic regression analysis. The cut point of CysC for DFU was also evaluated by receiver operating characteristic (ROC) curve. The prevalence of coronary artery disease, diabetic nephropathy (DN), and DFU dramatically increased with CysC (*P* < 0.01) in CysC quartiles. Multivariate logistic regression analysis indicated that the significant risk factors for DFU were serum CysC, coronary artery disease, hypertension, insulin use, the differences between supine and sitting TcPO_2_, and hypertension. ROC curve analysis revealed that the cut point of CysC for DFU was 0.735 mg/L. Serum CysC levels correlated with DFU and severity of tissue loss. Our study results indicated that serum CysC was associated with a high prevalence of DFU in Chinese T2D subjects.

## 1. Introduction

Diabetes mellitus is a complex metabolic disease that poised huge challenge to healthcare systems across the globe. Despite increased efforts in prevention and improved understanding of the condition, there is still an upward spike in prevalence rate and a paradigm shift towards developing countries in the recent decades [1]. Complications of tissue loss in people with diabetes have been identified as a major cause of hospitalization and nontraumatic lower extremity amputation [2, 3]. Diabetic foot ulceration (DFU) correlated to failure of healing, longer length of inpatient stay, and increased mortality, with adjusted relative ratio for length of stay and adjusted odds ratio for inpatient mortality was 2.01 and 1.31, respectively [4, 5]. Therefore, early diagnosis and appropriate management of DFU play a crucial role in amputation prevention and reduction of mortality in diabetic population. Moreover, the underlying mechanism of DFU is poorly comprehended with few possible biomarkers associating DFU prediction. A reliable predictive biomarker to identify and diagnose risk for DFU and its potential severity remains elusive yet. The severity and prognosis of DFU have a close relationship with renal insufficiency, peripheral artery disease (PAD), and neuropathy [6], consequently making it possible for early screening and identification of DFU biomarker related to above diseases.

Serum cystatin C (CysC) is a small molecular protein that produced by human nucleated cells and belongs to type 2 cystatin superfamily that functions as reversible competitive inhibitors of cysteine proteinase. CysC is freely filtered by the glomerulus and almost completely reabsorbed in the distal tubule without tubular secretion. Based on these reasons, CysC has been shown to be more reliable biomarker of renal function assessment compared with creatinine. It has been recognized as endogenous marker and noninvasive risk predictor of DN [7]. Apart from renal function, CysC was found to have an associative relationship and strong potential predictors, with PAD and diabetic peripheral neuropathy (DPN) [8, 9]. In our previous study, CysC level was higher in type 2 diabetes (T2D) with DPN than non-DPN, but no significant difference to blood creatinine or 24-hour urinary protein (24 h UP) between different groups was found [10]. Whether CysC has association for DFU in T2D subjective is still unknown. Therefore, in this study, we investigate the association between serum CysC and T2D patients with DFU.

## 2. Methods

In this study, we enrolled a total of 411 subjects with T2D admitted to a university hospital, between October 2011 to September 2012. The diagnosis of diabetes and its complications including DPN, DN, and diabetic retinopathy (DR) were based on the diagnostic criteria recommended by the ADA in 2010 [11]. DFU is defined as “ulceration of the foot (distally from the ankle and including the ankle) associated with neuropathy and different grades of ischemia and infection” according to the World health Organization [12]. DFU was categorized with the Wagner grade (from grade 0 to grade 5) and the society for vascular surgery lower extremity threatened limb classification system based on Wound, Ischemia, and Foot Infection (WIfI) [13, 14]. The diagnosis of hypertension was based on the diagnostic criteria recommended by the World Health Organization and the International Society of Hypertension in 1999 [15]. The patients with type 1 diabetes, other specific types of diabetes, and acute non-DFU diabetic complications were excluded from participation in the study. The study was approved by the ethics committee of the Southwest Hospital, and all of the subjects provided written informed consent.

All enrolled patients received ongoing medications for diabetes, hypertension, and hyperlipidemia throughout the duration of the study, except when overnight fasting blood samples were collected. Demographic information on age, sex, diabetes duration, hereditary history, systolic and diastolic blood pressure, smoking and drinking history, hypertension, coronary artery disease (CAD), and other diabetic complications was recorded for all participants. The systolic ankle and arm pressures were measured three times using the Doppler technique (Diabetic Foot Assessment Kit, Huntleigh Healthcare, UK). Ankle/brachial index (ABI) was determined by dividing the average of three systolic ankle pressures by the systolic brachial pressure. The transcutaneous oxygen pressure (TcPO_2_) was measured with the TCM 400 system (Radiometer, Copenhagen, Denmark) at an electrode temperature of 44°C. TcPO_2_ measurements were performed at the dorsum of the right foot proximal to the first and second toes, in both supine and sitting position with their legs hanging down. The average calibration period was 20 min. Measurements of TcPO_2_ in both supine and sitting positions and difference between sitting and supine position were expressed in mmHg, and the average of the last 5 min was used in the analysis.

Overnight fasting blood samples were collected and transported to the medical laboratory of Southwest Hospital. Fasting blood glucose (FBG) was assayed using the glucose oxidase method. Glycated hemoglobin (HbA1c) was measured by chromatography. Hemoglobin level was obtained using a hematology analyser. Serum uric acid (UA), creatinine, potassium, calcium, albumin, total cholesterol (TC), triglycerides (TG), low-density lipoprotein C (LDL-C), and high-density lipoprotein C (HDL-C) concentrations were determined with enzymatic method. 24 h UP, 24-hour urinary microalbumin (24 h UM), and serum CysC concentration were measured by immunoturbidimetry.

The experimental data were analyzed using SPSS software version 19.0 (IBM, IL, USA). Continuous variables are presented as the means ± standard deviations, and differences between groups were evaluated by Student's *t*-test or Analysis of Variance. Categorical variables are presented as frequency percentage and intergroup comparison using the chi-square test. The data were subjected to normal distribution analysis using the Kolmogorov-Smirnov test before statistical analysis. The association between CysC and clinical characteristics was performed by Spearman's correlation analysis. For multivariate analysis, we used a forward likelihood ratio logistic regression to identify the independent risk factors for DFU. Receiver operating characteristic (ROC) curve analyses were performed to determine the sensitivity and specificity of CysC for predicting DFU. The cut-off values were obtained from the ROC curves. A *P* value of <0.05 was considered statistically significant for all of the analyses.

## 3. Results

The clinical characteristics between patients with and without DFU were showed in Table 1. Compared with patients without DFU, DFU patients were significantly older and higher prevalence of DN and CAD (*P* < 0.01). Serum creatinine (*P* < 0.001), 24 h UP (*P* < 0.001), and 24 h UM (*P* < 0.001) were higher in DFU group, while DBP (*P* < 0.05), hemoglobin (*P* < 0.001), serum calcium (*P* < 0.001), serum albumin (*P* < 0.001), TG (*P* < 0.05), HDL-C (*P* < 0.05), ABI (*P* < 0.001), sitting position TcPO_2_ (*P* < 0.001), and supine position TcPO_2_ (*P* < 0.001) were lower in DFU patients. There was no significant difference in gender, diabetes duration, smoking, drinking, diabetes hereditary, DPN, DR, SBP, serum UA, potassium, TC, LDL-C, and difference between sitting and supine of TcPO_2_ in two groups (all *P* > 0.05).

Spearman's correlation analysis showed that serum CysC was closely associated with DFU (*r* = 0.347; *P* < 0.001), diabetes duration (*r* = 0.156; *P* = 0.002), gender (*r* = 0.189; *P* < 0.001), smoking (*r* = 0.118; *P* = 0.016), CAD (*r* = 0.166; *P* = 0.001), DBP (*r* = −0.224; *P* < 0.001), hemoglobin (*r* = −0.264; *P* < 0.001), UA (*r* = 0.360; *P* < 0.001), creatinine (*r* = 0.688; *P* < 0.001), potassium (*r* = 0.134; *P* = 0.007), albumin (*r* = −0.334; *P* < 0.001), 24 h UP (*r* = 0.109; *P* = 0.05), 24 h UM (*r* = 0.257; *P* < 0.001), sitting position TcPO_2_ (*r* = −0.294; *P* < 0.001), supine position TcPO_2_ (*r* = −0.259; *P* < 0.001), and ABI (*r* = −0.122; *P* = 0.013). In DFU patient population, CysC was closely associated with the Wagner grade (*r* = 0.228; *P* = 0.029), but no association with WIfI classification system. Furthermore, the serum CysC level gradually increased with the increase of Wagner grade 0 to 5.

Table 2 shows the odds ratios from the multivariate logistic regression model, in which DFU was the dependent variable and the risk factors mentioned above were covariates. The results showed that the significant risk factors included serum CysC level (*P* = 0.003; OR = 4.828), the presence of CAD (*P* = 0.005; OR = 3.566), insulin use (*P* = 0.010; OR = 2.605), the differences between supine and sitting of TcPO_2_ (*P* = 0.001; OR = 1.076), and the presence of hypertension (*P* = 0.023; OR = 1.021).

**Figure 1:**
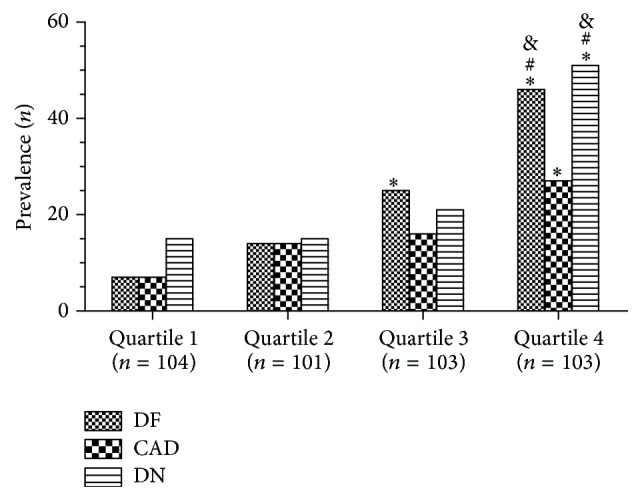
Prevalence of diabetic foot, coronary artery disease, and diabetic nephropathy by CysC quartiles. Quartile 1, CysC: ≤0.58 mg/L; Quartile 2, CysC: 0.58–0.7 mg/L; Quartile 3, CysC: 0.7–0.87 mg/L; Quartile 4, CysC: ≥0.87 mg/L. ^*∗*^ *P* < 0.001, compared with the first quartile; ^#^ *P* < 0.001, compared with the second quartile; ^&^ *P* < 0.001, compared with the third quartile. DFU, diabetic foot; CAD, coronary artery disease; DN, diabetic nephropathy.

From Table 3, the concentration of CysC increased with increasing age, serum UA, and creatinine concentration but decreased with DBP, albumin, sitting position TcPO_2_, and supine position TcPO_2_. As shown in Figure 1, the prevalence of CAD, DN, and DFU increased with CysC concentration, and the incidence of DFU in patients with the highest CysC quartile dramatically increased compared to other three quartile groups. The similar trend was also observed in patients with highest creatinine quartile.

**Figure 2:**
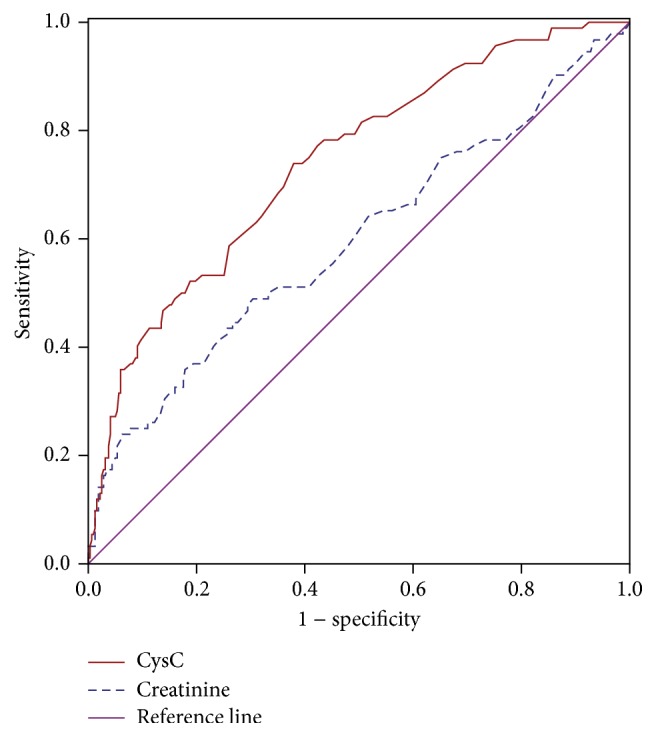
ROC analysis of CysC and creatinine to indicate diabetic foot for all diabetes participants. For CysC, AUC = 0.740, 95% confidence interval, 0.683–0.798; identified cut-off value = 0.735 mg/L; Youden index = 0.360; sensitivity: 73.9%; specificity: 62.1%.

**Figure 3:**
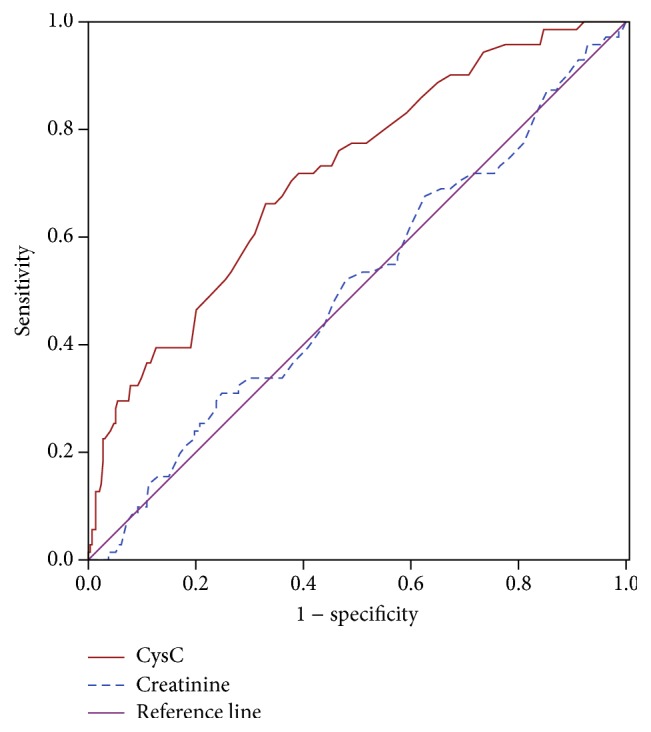
ROC analysis of CysC and creatinine to indicate DPN for nonclinical renal dysfunction patients. For CysC, AUC = 0.715; 95% confidence interval, 0.649–0.781; identified cut-off value = 0.735 mg/L; Youden index = 0.332; sensitivity, 66.2%; specificity, 67.0%.

ROC curve analysis was performed to verify the diagnostic accuracy of CysC and creatinine for DFU. The CysC has a better diagnostic value than creatinine. The area under the curve (AUC) of the CysC was 0.740 (95% confidence interval, 0.683–0.798) and had an optimal cut point value (0.735 mg/L) for the identification of DFU, with a sensitivity of 73.9% and a specificity of 62.1% in all patients (Figure 2). We also analyzed the ROC curve in nonclinical renal dysfunction diabetic patients (defined by serum creatinine < 97 *μ*mol/L) in order to exclude the influence by overt renal disease. The AUC of the CysC was 0.715 (95% confidence interval, 0.649–0.781) and also had an optimal cut point value (0.735 mg/L) for the identification of DFU in all diabetic patients without overt renal dysfunction, with a sensitivity of 66.2% and a specificity of 67.0% (Figure 3). However, serum creatinine has no such diagnostic ability.

## 4. Discussion

This cross-sectional study involving 411 Chinese T2D hospitalized patients is the first to investigate the relationship between CysC levels and DFU. Serum CysC level in DFU group was much higher than non-DFU group, and the CysC level was strongly and independently with the prevalence of DFU disease. Furthermore, the serum CysC level over 0.735 mg/L indicated a dramatically increased risk of DFU, but this relationship did not exist in creatinine. Therefore, serum CysC was considered to be a probable marker for DFU in T2D populations.

PAD is one of the most critical factors for prevalence of DFU in T2D, induced by atherosclerosis, hardening, and narrowing of vessel walls leading to arterial occlusion. The traditional risk factors of PAD include advanced age, smoking, hypertension, inadequate control of blood sugar, and lipid disorder. In this study, the prevalence of DFU was higher in T2D patients with age, consistent with previous studies [16, 17]. In comparison with blood lipid components between DFU and non-DFU patients, we found that the only significant difference was HDL-C concentration. Drawing similarity with a previous meta-analysis study, decreased HDL-C had a significant association with DFU susceptibility, but no significant associations were found between DFU and LDL-C, TC, or TG levels [18]. The percentage of smoking, hypertension, and HbA1c were higher in DFU than non-DFU even if all of them had no significant difference. Compared to non-DFU patient, DBP level was lower in DFU patient, consistent with a previous study [8]. Unexpectedly, beside the traditional risk factors, we also found that insulin use was one of significant risk factors for DFU. In a cross-sectional study of 62,681 diabetic patients, insulin use also was found to be one of significant risk factors of DFU with odds ratio and 95% confidence interval at 4.69 (4.28–5.14) [17]. It is necessary to further clarify the possible underlying mechanism with a prospective study in order to exclude some confounders besides insulin utility.

To examine the relationship between DFU and diabetic renal complication, a retrospective study of 44,917 hospitalized diabetic patients in Singapore found that renal disease had significant higher rates of diabetic lower extremity amputation compared to diabetic patients without renal disease. After logistic regression analyses, renal disease was one of significant predictors of diabetic lower extremity amputation (renal disease: odds ratio 3.2, 95% confidence interval 2.8–3.6) after adjustment for age, gender, and year of discharge [19]. In another observational study of diabetic patients, the result revealed that the stage of chronic kidney disease strongly associated with DFU ulcer or lower extremity amputation, independent of PAD [20]. In line with findings of the above-mentioned studies, we also found that DN was close association with prevalence of DFU in our study.

Serum CysC has slowly gained more widespread acceptance as an alternative endogenous filtration marker. Serum CysC concentrations are less influenced by muscle mass and diet than creatinine [21]. Furthermore, serum CysC has been proved a strong relationship with cardiovascular disease (CVD) and peripheral vascular disease. Serum CysC was not only an indicator for renal dysfunction in diabetic patients with overt nephropathy but also a predictor for cardiovascular events in diabetic patients without nephropathy [22]. In this study, we found that CAD has contributed considerably to the prevalence of DFU and was one of independent risk factors for DFU. Furthermore, CysC level was found to be correlated significantly to insulin resistance and biomarkers reflecting inflammation, independent of renal function [23]. These components may have a role in addition to that of eGFR in explaining the link between CysC and CVD in T2D patients.

Comorbid conditions associated with DFU often include PAD, DPN, and cutaneous wound infection. In this study, ABI was slightly decreased in DFU patients. However ABI is often falsely elevated in people with diabetes because of medial calcinosis. Instead, the difference between supine and sitting TcPO_2_ was used and appeared to be associated as risk factors of DFU. For this reason, in the recently established WIfI classification wound system, to identify lower extremity ischemia, TcPO_2_ was chosen to be one of acceptable alternatives rather than ABI if toe pressure is unavailable [13]. A recent systematic review study revealed that even though TcPO_2_ may have problems with reproducibility in some reports, TcPO_2_ was more useful in predicting ulcer healing and amputation than ABI [24]. This is plausible, since it is a more direct measure of tissue perfusion rather than hemodynamics. PAD, CAD, and hypertension have common disease pathogenesis pathways such as insulin resistance, atherosclerosis, and lipid dysfunction. Besides the influence of CysC on CVD, there is also a strong and independent association between CysC and limb arterial disease was found in a study of Chinese diabetic population [8]. Arpegård et al. found that CysC may be an independent marker of atherosclerotic disease apart from its correlation to kidney [25]. Like serum CysC, peroxisome proliferator activator receptor *γ* and advanced oxidation protein product are found to be closely related to atherosclerosis in T2D patients [26]. Furthermore, hyperglycemia causes blood hypercoagulable state. Cytokine secreted from platelet has an effect on prognosis of atherosclerosis. CysC was downregulated by miR-92a derived from platelets, while a miR-92a inhibitor upregulated CysC expression. miR-92a directly entered into endothelial cells via cell interactions and caused atherosclerosis in diabetic lower limb ischemia [27].

As mentioned before, CysC is a cysteine protease inhibitor that may influence immune response and induce infection. In a study of 30,329 adults during 10-year observation period, Powell et al. found that elevated CysC was associated with increased long-term rates of community-acquired sepsis, independent of abnormal estimated glomerular filtration rate, albumin-to-creatinine ratio, and high sensitivity C-reactive protein [28]. In another study of 5,142 cardiovascular health study participants, the authors found that serum CysC may suggest additional factors beyond kidney function that were associated with infection risk [29]. Immunity impairment can predispose diabetic patients to DFU infection. Signs of infection include increased redness, swelling, pain, and purulent drainage, along with an elevated white blood cell count and markers of inflammation [13, 30]. CysC was significantly associated with inflammation markers, such as white blood cell and C-reactive protein. Even after adjusting for confounding factors, the significant association still remained [31, 32]. In this study, CysC levels had a significant linear association with Wagner classification system, demonstrating a possible relationship to severity of DFU infection. Putting together, CysC may promote and induce DFU infection, but the mechanism needs further validated.

There are some limitations in our study. First of all, the number of patients with DFU was relatively small, so that it is hard to investigate the association between serum CysC and DFU according to different groups, including neuropathic ulcer or ischemic ulcer or neuropathic-ischemic ulcer. Secondly, our findings may not be applicable to other types of diabetes with different pathophysiology. At last, the sample population of this study only includes Chinese patients from local Chongqing provinces and thus may not be representative to other ethnic groups in the general population pool in China and beyond. We look forward to further works improving upon this methodology in other countries.

In conclusion, this study brings new insight to our traditional view of CysC as biomarker of renal function and, arguably, other end-stage complications of diabetes as well. Our findings suggest that serum CysC was closely associated with the prevalence and severity of DFU. The measurement of CysC concentration is of importance and value for screening out the T2D patients with high risk of foot disease, in order to salvage lower limb. Therefore, serum CysC may be a useful potential biomarker for DFU in T2D patient. However, future longitudinal study should be performed in order to exactly observe the association between CysC level and outcome of DFU. In addition, further basic research of underlying mechanisms between CysC and DFU may be useful to explore novel therapeutic strategy for DFU prevention and lower limbs salvage.

## Figures and Tables

**Table 1:** Comparison of clinical characteristics between patients with and without diabetic foot.

	With DFU	Without DFU	*P* value
*N* (male/female)	92 (55/37)	319 (181/138)	0.603
Age (ys)	66.42 ± 11.64	60.13 ± 10.60	<0.001
Duration (ys)	9.45 ± 7.69	8.07 ± 5.92	0.066
Smoking (%)	35.87	27.59	0.125
Drinking (%)	22.83	19.44	0.475
Hereditary (%)	22.83	31.35	0.114
IU (%)	77.55	49.40	<0.001
DPN (%)	70.65	76.49	0.254
DR (%)	38.00	25.58	0.083
DN (%)	36.46	22.92	0.008
HTN (%)	56.52	57.68	0.843
CAD (%)	29.35	11.60	<0.001
SBP (mmHg)	134.52 ± 19.74	131.62 ± 17.70	0.076
DBP (mmHg)	77.07 ± 12.01	80.26 ± 11.44	0.020
Hemoglobin (g/L)	113.13 ± 17.17	128.16 ± 17.11	<0.001
UA (*μ*mol/L)	283.08 ± 108.83	286.18 ± 89.47	0.278
Cr (*μ*mol/L)	82.22 ± 51.49	63.32 ± 26.39	<0.001
Potassium (mmol/L)	3.89 ± 0.57	3.87 ± 0.40	0.595
Calcium (mmol/L)	2.18 ± 0.20	2.25 ± 0.13	<0.001
Albumin (g/L)	34.40 ± 6.13	39.88 ± 4.77	<0.001
TC (mmol/L)	4.49 ± 1.25	4.63 ± 1.09	0.297
TG (mmol/L)	1.56 ± 0.93	1.89 ± 1.34	0.029
LDL-C (mmol/L)	2.67 ± 0.79	2.74 ± 0.71	0.451
HDL-C (mmol/L)	1.07 ± 0.38	1.16 ± 0.31	0.035
24 h UP (mg)	1117.62 ± 652.22	337.21 ± 189.57	<0.001
24 h UM (mg)	47.73 ± 28.25	15.62 ± 8.25	<0.001
ABI	1.01 ± 0.26	1.09 ± 0.11	<0.001
SiTcPO_2_	47.11 ± 15.81	64.81 ± 10.29	<0.001
SuTcPO_2_	27.29 ± 13.63	44.18 ± 11.83	<0.001
DSS TcPO_2_	20.24 ± 10.08	20.62 ± 7.36	0.686

Data represent means ± SD or frequency (percentage).

IU: insulin use; DPN: diabetic peripheral neuropathy; DR: diabetic retinopathy; DN: diabetic nephropathy; HTN: hypertension; CAD: coronary artery disease; SBP: systolic blood pressure; DBP: diastolic blood pressure; UA: serum uric acid; Cr: serum creatinine; TC: total cholesterol; TG: triglyceride; LDL-C: low-density lipoprotein cholesterol; HDL-C: high-density lipoprotein cholesterol; 24 h UP: 24-hour urinary protein; 24 h UM: 24-hour urinary microalbumin; ABI: ankle/brachial index; SiTcPO_2_: sitting position transcutaneous oxygen pressure; SuTcPO_2_: supine position TcPO_2_; DSS TcPO_2_: the differences between supine and sitting of TcPO_2_.

**Table 2:** Risk factors for DFU by multivariate logistic regression analysis.

	*B*	SE	Wald	*P*	OR	95% CI for OR	
						Lower	Upper
CysC	1.574	0.529	8.853	0.003	4.828	1.711	13.620
CAD	1.271	0.452	7.912	0.005	3.566	1.470	8.648
IU	0.957	0.371	6.646	0.010	2.605	1.258	5.394
DSS TcPO_2_	0.073	0.021	11.767	0.001	1.076	1.032	1.122
HTN	0.021	0.009	5.171	0.023	1.021	1.003	1.039

CI: confidence interval; CysC: serum cystatin C; CAD: coronary artery disease; IU: insulin use; DSS TcPO_2_: the differences between supine and sitting transcutaneous oxygen pressure; HTN: hypertension.

**Table 3:** Comparison of clinical characteristics and experiment indices among cystatin C quartiles.

Cystatin C	≤0.58 mg/L	0.58–0.7 mg/L	0.7–0.87 mg/L	≥0.87 mg/L	*P* value
Age (ys)	53.58 ± 9.21	60.14 ± 8.62	64.17 ± 9.98	68.31 ± 11.01	<0.001
Duration (ys)	7.67 ± 6.05	7.79 ± 5.55	8.48 ± 7.07	9.56 ± 6.62	0.126
Sex (M/F)	104 (45/59)	101 (61/40)	103 (58/45)	103 (72/31)	<0.001
Smoking (%)	23.08	29.70	25.24	38.83	0.041
Drinking (%)	17.31	20.79	17.48	25.24	0.447
Hereditary (%)	42.30	26.73	19.42	29.13	0.003
DPN (%)	69.23	73.27	77.67	80.58	0.248
DR (%)	25.00	26.73	33.98	30.10	0.501
IU (%)	42.31	50.50	56.31	70.87	<0.001
FBG (mmol/L)	8.93 ± 2.77	8.28 ± 2.61	8.87 ± 3.29	8.70 ± 4.00	0.472
SBP (mmHg)	132.41 ± 17.28	131.41 ± 16.34	131.86 ± 17.87	133.39 ± 21.11	0.880
DBP (mmHg)	83.22 ± 11.47	80.95 ± 10.57	78.52 ± 10.90	75.48 ± 12.22	<0.001
ABI	1.08 ± 0.09	1.09 ± 0.13	1.06 ± 0.15	1.05 ± 0.23	0.356
Hemoglobin (g/L)	128.59 ± 15.73	129.83 ± 15.76	124.21 ± 16.85	116.62 ± 21.18	<0.001
UA (*μ*mol/L)	243.21 ± 69.01	270.24 ± 79.82	290.64 ± 90.93	337.96 ± 106.52	<0.001
Creatinine (*μ*mol/L)	48.94 ± 13.08	56.62 ± 14.71	63.16 ± 17.38	101.45 ± 49.49	<0.001
CysC (mg/L)	0.51 ± 0.07	0.64 ± 0.03	0.78 ± 0.05	1.26 ± 0.45	<0.001
Calcium (mmol/L)	2.25 ± 0.12	2.24 ± 0.13	2.24 ± 0.19	2.21 ± 0.15	0.203
Potassium (mmol/L)	3.85 ± 0.38	3.83 ± 0.36	3.81 ± 0.52	4.00 ± 0.49	0.012
HbA1c (%)	8.33 ± 2.13	7.77 ± 1.96	7.96 ± 1.96	7.82 ± 1.86	0.168
Albumin (g/L)	40.89 ± 4.66	39.21 ± 4.88	38.19 ± 5.22	35.77 ± 6.23	0.020
SiTcPO_2_	65.70 ± 11.30	63.58 ± 12.19	60.27 ± 12.99	53.84 ± 15.73	<0.001
SuTcPO_2_	45.72 ± 12.09	42.53 ± 13.94	39.41 ± 13.76	33.92 ± 14.08	<0.001
DSS TcPO_2_	20.09 ± 7.25	21.02 ± 8.35	20.85 ± 8.69	20.20 ± 7.88	0.800
TC (mmol/L)	4.76 ± 0.88	4.63 ± 1.06	4.41 ± 1.14	4.61 ± 1.36	0.177
TG (mmol/L)	1.90 ± 1.36	1.90 ± 1.61	1.66 ± 0.85	1.79 ± 1.14	0.479
LDL-C (mmol/L)	2.81 ± 0.61	2.73 ± 0.65	2.61 ± 0.75	2.74 ± 0.86	0.278
HDL-C (mmol/L)	1.18 ± 0.31	1.15 ± 0.31	1.15 ± 0.34	1.07 ± 0.33	0.091

Data represent means ± SD or frequency (percentage).

DPN: diabetic peripheral neuropathy; DR: diabetic retinopathy; IU: insulin use; FBG: fasting blood glucose; SBP: systolic blood pressure; DBP: diastolic blood pressure; ABI: ankle/brachial index; UA: serum uric acid; CysC: serum cystatin C; HbA1c: glycated hemoglobin; SiTcPO_2_: sitting position transcutaneous oxygen pressure; SuTcPO_2_: supine position TcPO_2_; DSS TcPO_2_: the differences between supine and sitting of TcPO_2_; TC: total cholesterol; TG: triglyceride; LDL-C: low-density lipoprotein cholesterol; HDL-C: high-density lipoprotein cholesterol.
